# Correlation Analysis between Serum Vitamin D Levels and Lower Extremity Macrovascular Complications in Individuals with Type 2 Diabetes Mellitus

**DOI:** 10.1155/2019/4251829

**Published:** 2019-11-28

**Authors:** Mengxue Yang, Jun Liu, Xue Zhou, Heyuan Ding, Jie Xu, Bo Yang, Bowen Sun, Dandan Xiao, Jie Yu, Qihai Gong

**Affiliations:** ^1^Department of Endocrinology, The Fifth People's Hospital of Shanghai, Fudan University, Shanghai 200240, China; ^2^Department of Endocrinology, Zunyi Medical University, Zunyi 563000, China; ^3^School of Public Health, Zunyi Medical University, Zunyi 563000, China; ^4^Key Laboratory of Basic Pharmacology of Ministry of Education and Joint International Research Laboratory of Ethnomedicine of Ministry of Education, Zunyi Medical University, Zunyi 563000, China

## Abstract

The correlation between serum 25-hydroxy vitamin D (25(OH)D) levels and lower extremity atherosclerotic disease and the predictive value of 25(OH)D for early-stage lower extremity atherosclerotic disease in patients with type 2 diabetes mellitus (T2DM) were explored. In total, 620 subjects (590 T2DM patients and 30 healthy subjects) completed a questionnaire. All subjects were divided into four groups according to serum 25(OH)D concentration quartile: Q1 (<12.18 ng/ml), Q2 (12.18~20.65 ng/ml), Q3 (20.65~31.97 ng/ml), and Q4 (>31.97 ng/ml). Participants were also divided into four groups based on the degree of lower extremity arteriostenosis: A1 (T2DM), A2 (T2DM with mild lower extremity vascular lesions (LEVL)), A3 (T2DM with moderate LEVL), and A4 (T2DM with severe LEVL). The incidence of lower extremity artery plaque was significantly higher in groups Q1 and Q2 than in group Q4 (both *P* < 0.05). The concentration of 25(OH)D was significantly lower in group A4 than in groups A1 and A2. Pearson correlation analysis revealed that the degree of lower extremity vascular stenosis was positively correlated with age, smoking, and HbA1c, CRP, and LDL-C levels and negatively correlated with 25(OH)D concentrations. Logistic regression analysis demonstrated that 25(OH)D concentrations exerted a protective effect against LEVL in T2DM patients. Serum 25(OH)D concentrations may be correlated with the incidence of macrovascular disease in T2DM patients. A low serum 25(OH)D concentration is an independent risk factor for lower extremity vascular pathological changes and acts as a prognostic index for lower extremity atherosclerotic disease.

## 1. Introduction

Diabetic atherosclerosis (AS), especially lower extremity macrovascular complications, which is the primary cause of lower extremity amputation is one of the most severe diabetic complications [1]. AS not only significantly reduces the survival rate of diabetic patients but also affects their quality of life. Early discovery of diabetic AS plays a crucial role in the prevention and treatment of this condition.

AS is a systemic disease of the whole body with local manifestations, and arterial plaques in the carotid arteries and peripheral arteries that are easy to detect can indirectly reflect the status of AS in the whole body. Currently, ultrasound, computed tomography angiogram (CTA), magnetic resonance angiography (MRA), digital subtraction angiography (DSA), and ankle-brachial index (ABI) are used to assess the state of the lower extremity arteries. However, these techniques are associated with complex examination procedures and high cost. Furthermore, these techniques require specialized equipment and professional operators and have many requirements in terms of techniques and equipment. Therefore, they are not proper for the early diagnosis and screening of AS in outpatient departments [2], and seeking an easily manipulated, effective, and relatively noninvasive method remains urgent.

Vitamin D is a secosteroid that is obtained from exposure to sunlight and through dietary sources, including food and supplements. Vitamin D is hydroxylated in the liver to form 25-hydroxyvitamin D (25(OH)D) and is further hydroxylated in the kidney to form 1,25-hydroxyvitamin D (1,25(OH)2D, calcitriol). Although 1,25(OH)2D is considered to be the active form of vitamin D, its level in the serum does not correlate with overall vitamin D status, whereas the 25(OH)D level is a more clinically relevant marker [3].

The main function of vitamin D is to regulate calcium and phosphorus metabolism, and it also plays roles in the immune, blood, and endocrine systems. Recent studies have found that vitamin D supplementation tends to exert varying degrees of influence upon the incidence and progression of multiple chronic diseases, such as depression, autoimmune diseases, infectious diseases, diabetic nephropathy, and cardiovascular diseases [4–7]. It has been demonstrated that a lack of 25(OH)D3 is likely to impact the expression and effect of macrophages and lymphocytes in atherosclerotic plaques and accelerate the chronic inflammatory responses of the arterial wall. Vitamin D3 is capable of exerting an anti-AS effect mediated by the vitamin D receptor [8]. To date, however, the role of 25(OH)D in T2DM patients with AS has only rarely been reported. Although Zhou et al. reported that a low level of 25(OH)D is significantly associated with the occurrence of T2DM complicated with lower extremity arterial disease (LEAD) [9], this study focused on only the association of vitamin D with T2DM complicated with noticeable LEADs and did not explore the preventive index of early lower extremity AS in diabetes patients.

Therefore, in this study, we detected serum 25(OH)D levels in T2DM patients with macrovascular complications and investigated the feasibility of using serum 25(OH)D levels as a predictor of preclinical lower extremity AS in T2DM patients. The association between serum 25(OH)D concentrations and the degree of lower extremity stenosis was further analyzed with the expectation of discovering risk factors for AS in T2DM patients. The results of this study may therefore provide a theoretical reference for the prevention and treatment of this condition.

## 2. Materials and Methods

### 2.1. Study Subjects

Five hundred and ninety T2DM patients, 303 males and 289 females with an average age of 53 ± 17 years, and healthy subjects with normal glucoregulation (*n* = 30) admitted to the affiliated hospital of Zunyi Medical College between September 2012 and October 2015 were recruited for this study. The healthy controls were age- and sex-matched with the patients. All participants performed a 75 g oral glucose tolerance test (OGTT). The diagnosis of T2DM was performed according to the 1999 World Health Organization (WHO) classification and diagnostic criteria of diabetes. The exclusion criteria were as follows: (1) those with a medical history of diabetes and use of hypoglycemic drugs; (2) those with type I diabetes mellitus, gestational diabetes mellitus, or specific types of diabetes mellitus; (3) those with acute complications of diabetes mellitus (such as diabetic ketoacidosis, hyperglycemia hyperosmotic state, and lactic acidosis); (4) those with serious infections or stress responses; (5) those with hepatic and renal insufficiency; (6) those with malignant tumors, connective tissue diseases, coronary heart diseases, and stroke; and (7) those who use medications that affect test results (such as contraceptive and statin drugs).

All participants were biologically unrelated Chinese Han individuals. Written informed consent was obtained from all included individuals. This study was approved by the Ethics Committee of Zunyi Medical College (approval no. (2012) 1-106).

### 2.2. Grouping

Based on the presence of lower extremity macrovascular complications, 460 participants were assigned to the simple T2DM group (227 males and 233 females) and 130 were assigned to the T2DM with lower extremity macrovascular complications group (66 males and 64 females). The incidence of hypertension, diabetic nephropathy, diabetic retinopathy, and smoking did not significantly differ between the two groups (all *P* > 0.05).

HP Color Doppler diagnostic equipment (HP Company, U.S.) was utilized to examine the lower extremity arteries (femoral artery, popliteal artery, anterior/posterior tibial artery, and foot dorsal artery). The detected lower extremity vascular complications were divided into four categories, including endarterial thickness, severity of AS, plaque, and stenosis. The grading was evaluated based upon the severity of vascular lesions [10]: 0 point for normal, 1 point for mild lesions, 2 points for moderate lesions, and 3 points for diffuse plaque or vascular occlusion for a total of 20 points. The severity of lesions was assessed based on the total score, and all participants were subject to the following grouping: (1) simple T2DM group—ultrasound examination revealed normal blood vessels of the lower extremities (evaluated as 0 points); and (2) T2DM with lower extremity vascular complications group, which was divided into three subgroups including the mild group (1-9 points), the moderate group (10-19 points), and the severe group (20 points).

Moreover, all patients were assigned into two groups according to the presence of blood glucose fluctuation: the blood glucose fluctuation group (the difference in blood glucose before and after meals > 2.8 mmol/l) and the glucostasis group (the difference in blood glucose before and after meals < 2.8 mmol/l).

### 2.3. Methods

#### 2.3.1. Questionnaire

All staff received unified training for the study. The questionnaire survey included years of education (≤6, 7-9, 10-12, and ≥13 years), smoking history, history of drinking tea or coffee, physical activity, work environment, and the use of calcium or vitamin D supplements.

#### 2.3.2. Observational Indices

*(1) Detection of Body Fat Parameters*. Body mass index (BMI) was used as a parameter: BMI = weight/height^2^ (kg/m^2^).

*(2) Detection of Biological Parameters*. Peripheral blood was taken with a vacutainer (BD, USA) when the participants had an empty stomach in the morning. The sample was centrifuged immediately, and detection was completed within 2 h. Venous blood samples were prepared, and the serum was separated before the measurement of serum chemerin (ELISA, R&D Systems Corporation, U.S., intrabatch coefficient of variation (CV) < 7.8% and interbatch CV < 9.8%), fasting blood glucose (glucose oxidase method), blood lipids, hepatic and renal function (automatic biochemistry analyzer), and HbA1c (HPLC). Venous blood samples were prepared for the measurement of blood glucose level 2 h after the OGTT.

### 2.4. Statistical Analysis

Measurement data are expressed as the means ± SDs. Measurement data between two groups were statistically compared using the *t*-test. Differences in measurement data among at least three groups were statistically analyzed by one-way ANOVA with the least significant difference (LSD) post hoc test. Pearson correlation analysis was performed when the data were normally distributed; otherwise, the Spearman rank correlation test was employed. Multiple stepwise regression analysis was utilized for multivariate analysis. SPSS 16.0 software was used for statistical analysis. *P* < 0.05 was considered statistically significant.

## 3. Results

### 3.1. General Data

The T2DM group showed significant differences in BMI, HbA1C, blood glucose before and after food intake, triglycerides (TGs), total cholesterol (TC), and *γ*-GGT compared with the same parameters in the normal control group (all *P* < 0.05, Table 1). Furthermore, these indices, except for *γ*-GGT, increased with the severity of lower extremity vascular lesions, showing significant differences (all *P* < 0.05, Table 2).

### 3.2. Questionnaire Survey Results

A split half reliability test was performed; the reliability coefficient of the questionnaire (*α*) was 0.82620, and the poststandardization reliability coefficient was 0.8690, which indicated a high reliability of the questionnaire. A total of 82% of the experts were satisfied or very satisfied with the contents of the questionnaire, and approximately 83% of the experts were satisfied with the structure of the questionnaire, indicating a high validity of the questionnaire.

In the young-age group, 22 subjects (22%) had normal serum concentrations of 25(OH)D, and 78 subjects (78%) had normal serum concentrations of 25(OH)D in the middle-age group. The nutritional status of vitamin D in the middle-age group was significantly better than that in the young-age group (*P* < 0.01). Among the 100 subjects, 10 (10%) received ≤6 years of education, 29 (29%) had an education level of 7-9 years, 33 (33%) had an education level of 10-12 years, 28 (28%) had an education level of ≥13 years, 40 subjects (40%) were smokers, 51 subjects (51%) had a habit of drinking tea or coffee, 24 subjects (24%) participated in outdoor activities for over 30 min daily, 62 subjects (62%) performed indoor work or studying, and 19 subjects (19%) took calcium or vitamin D supplements.

The effects of years of education on serum 25(OH)D levels were as follows: the serum 25(OH)D levels in patients receiving ≥13 years of education were significantly lower than those in their counterparts with <6, 7-9, and 10-12 years of education (*P* = 0.042). Among those with ≥13 years of education, 38% were professionals and technicians, 22% were employees of party and government organizations and public institutions, and 6% were students. The serum 25(OH)D level was negatively correlated with years of education (*r* = −0.385, *P* < 0.05).

### 3.3. Comparison of Serum 25(OH)D Levels

The T2DM with lower extremity vascular complications group was divided into three subgroups, including the mild group (1-9 points, *n* = 53, 27 males and 26 females), the moderate group (10-19 points, *n* = 40, 22 males and 18 females), and the severe group (20 points, *n* = 37, 17 males and 20 females).

In the hospital of the current team, the reference range of 25(OH)D is 20-32 ng/ml; therefore, all subjects were divided into the following four groups according to the quartile of serum concentration of 25(OH)D: group Q1 (<12.18 ng/ml, *n* = 32), group Q2 (12.18-20.65 ng/ml, *n* = 40), group Q3 (20.65-31.97 ng/ml, *n* = 28), and group Q4 (>31.97 ng/ml, *n* = 30).

**Figure 1:**
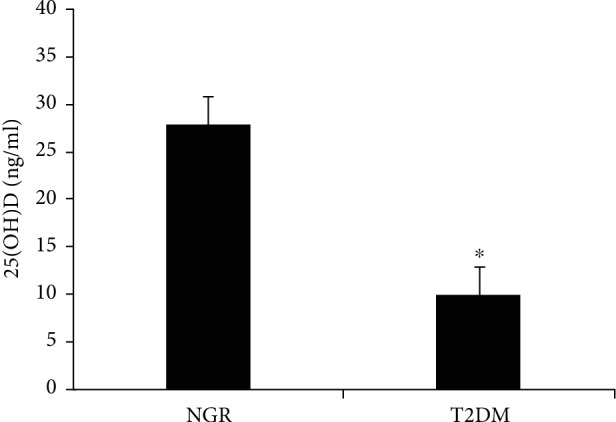
Comparison of serum 25(OH)D levels among different glucose regulation groups. NGR: normal glucose regulation; T2DM: type 2 diabetes mellitus; ^∗^*P* < 0.05 vs. NGR.

A comparison of serum 25(OH)D levels among different glucoregulation groups is illustrated in Figure 1. After BMI adjustment, the serum 25(OH)D level in the T2DM group was lower than that in the NGR group.

**Figure 2:**
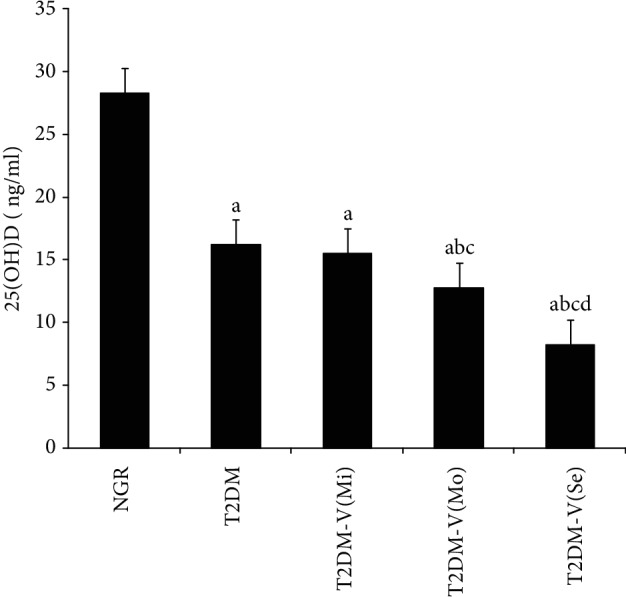
Comparison of serum 25(OH)D levels between groups with different degrees of lower extremity vascular disease. NGR: normal glucose regulation; T2DM: type 2 diabetes mellitus alone; T2DM-V(Mi): type 2 diabetes mellitus-vascular diseases (mild); T2DM-V(Mo): type 2 diabetes mellitus-vascular diseases (moderate); T2DM-V(Se): type 2 diabetes mellitus-vascular diseases (severe). ^a^*P* < 0.05 vs. NGR; ^b^*P* < 0.05 vs. T2DM; ^c^*P* < 0.05 vs. T2DM-V(Mi); ^d^*P* < 0.05 vs. T2DM-V(Mo).

The comparison of 25(OH)D levels among the three subgroups of individuals with T2DM with lower extremity vascular complications is shown in Figure 2. The severity of stenosis in the three subgroups was 15.5 ± 5.2, 12.8 ± 5.6, and 8.2 ± 4.5 ng/ml. Serum 25(OH)D levels in the moderate and severe stenosis subgroups of individuals with T2DM with lower extremity vascular complications were significantly lower (16.2 ± 8.8 ng/ml) than those in the simple T2DM and NGR groups (28.3 ± 6.9 ng/ml) (*P* < 0.05). Serum 25D levels in the moderate and severe stenosis groups were significantly lower than those in the mild stenosis group (*P* < 0.05). Moreover, the serum 25D level in the simple T2DM group was considerably lower than that in the NGR group (*P* < 0.05). The serum 25D level in the moderate group was significantly lower than that in the severe group (*P* < 0.05). Serum 25D levels in the mild and simple T2DM groups did not significantly differ (*P* > 0.05).

**Figure 3:**
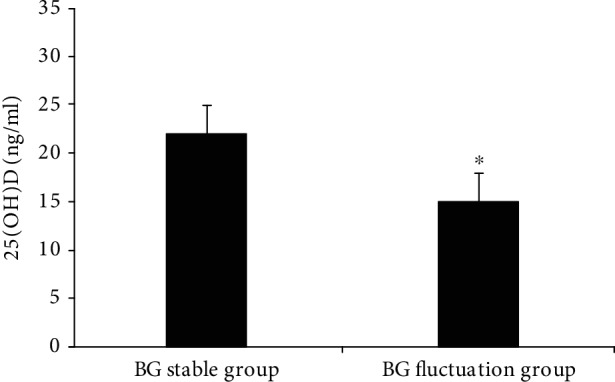
Comparison of serum 25(OH)D in the blood glucose fluctuation group and the stable blood glucose group. BG stable group: stable blood glucose group; BG fluctuation group: blood glucose fluctuation group. *P* < 0.05 vs. the stable blood glucose group.

A comparison of serum 25(OH)D levels between the blood glucose fluctuation and glucostasis groups is illustrated in Figure 3. The level of serum 25(OH)D in the blood glucose fluctuation group was significantly lower than that in the stable blood glucose group (15.23 ± 8.12 ng/ml and 22.06 ± 10.58 ng/ml, *P* < 0.05).

### 3.4. Comparison of the Low-Concentration Serum 25(OH)D Group with the High-Concentration Group

The incidence of lower extremity artery plaque; degree of arteriostenosis; course of diabetes; and the levels of fasting plasma glucose (FPG), HbA1c, and CRP differed significantly between the low- and high-concentration serum 25(OH)D groups (all *P* < 0.05). The incidence of lower extremity artery plaque in group Q1 (55.0% (84/152)) and group Q2 (49% (76/156)) was significantly higher than that in group Q4 (46.0% (71/155)) (both *P* < 0.05), whereas it did not significantly differ from that in group Q3. The incidence of lower extremity artery plaque in group Q1 was significantly higher than that in the other groups (all *P* < 0.05).

### 3.5. Correlation Analysis

Correlation analysis revealed that the severity of lower extremity angiostenosis was positively correlated with age, smoking, HbA1c, CRP, and LDL-C (*r* = 0.42, 0.48, 0.65, 0.28, and 0.55, respectively, all *P* < 0.05) and negatively correlated with 25(OH)D concentration (*r* = −0.269, *P* < 0.05). The score for lower extremity macroangiopathy was used as the dependent variable, and age, sex, the course of the disease, BMI, and medication (such as drugs for glucose reduction and lipid regulation) were introduced into multivariable regression analysis. The results showed that HbA1c and the 25(OH)D concentration were independently correlated with lower extremity macroangiopathy (both *P* < 0.05, Table 3).

## 4. Discussion

Vascular endothelial inflammatory response has been widely regarded as an essential factor involved in the incidence of AS [10]. However, the factor responsible for triggering and maintaining the inflammatory response remains to be determined. In addition to endothelial cells, multiple inflammatory cells also participate in the incidence and progression of AS, and monocytes account for approximately 80% of the participation. Peripheral blood monocytes migrate into the arterial endothelial space and differentiate into macrophages, which is the earliest event in the incidence of AS. Vitamin D receptors (VDRs) are expressed on monocytes/macrophages. Through mediation by monocyte/macrophage VDRs, vitamin D is capable of regulating the immune response, enhancing IL-10 levels, reducing NF-*κ*b activity, and decreasing the production of IL-6, IL-12, and THF-*α*, thereby alleviating the severity of AS [11, 12].

Vitamin D not only plays a vital role in calcium and phosphorus metabolism and bone calcification but also exerts a widespread effect on various types of tissues and cells. Recent research has demonstrated that vitamin D is significantly associated with the incidence of diabetic cardiovascular diseases. Vitamin D is able to cause AS and cardiovascular diseases via a variety of underlying mechanisms, which probably include the activation of the renin-angiotensin-aldosterone system, the increase in insulin resistance, and the increase in parathormone levels, resulting in left ventricular hypertrophy, metabolic syndrome, and systematic inflammation, thereby increasing the incidence of AS and cardiovascular events [13].

It has been proven that serum 25(OH)D levels are correlated with age [14, 15]. In this study, the serum 25(OH)D level in the young-age group was significantly lower than that in the middle-aged group, which is consistent with previous findings and is probably because 62% of the middle-aged population seldom exercised and had adverse lifestyle habits, such as smoking and drinking tea or coffee. In addition, serum 25(OH)D levels in the population with >13 years of education and possessing a doctoral degree were considerably lower in this study, possibly because the majority of this population were professionals, employees of party and government organizations, or students, and they seldom participated in physical activity and had unhealthy lifestyle habits, such as smoking and drinking tea and coffee. Currently, aging, smoking, and high levels of HbA1c and LDL-c have been recognized as major factors contributing to the incidence of AS. However, the relationship between vitamin D and the incidence of AS remains to be elucidated.

Multiple investigations have reported that vitamin D levels were associated with the incidence of AS [16–19], whereas other scholars argued that vitamin D levels were not correlated with macrovascular complications [1]. Other studies found that supplementation with vitamin D and its analogs most likely alleviated diabetic macrovascular complications [20]. In a mouse experiment, the administration of vitamin D- and cholesterol-enriched diets significantly quickened the progression of AS [21].

In this study, serum 25(OH)D levels were generally lower in T2DM patients. Moreover, the serum 25(OH)D level gradually decreased along with the aggravation of lower extremity vascular complications, prompting the idea that the serum 25(OH)D level is probably correlated with lower extremity vascular complications in T2DM patients, which is consistent with previous findings [9, 22, 23]. Previous studies demonstrated that T2DM, inflammation, and vitamin D deficiency were correlated with each other. Our preliminary investigation also found microinflammation in patients with T2DM and uremia complicated with diabetic nephropathy (DN). The serum 25(OH)D3 concentration was generally reduced in both T2DM and uremia patients with DN. Additionally, T2DM patients presented with a lower serum 25(OH)D3 level than uremia patients with DN [24].

Our present study indicated that the progression of diabetes mellitus, FPG, HbA1c, and CRP levels differed significantly between the groups with low and high serum 25(OH)D concentration (all *P* < 0.05), which is consistent with previous findings. The serum 25(OH)D level in patients with plaque was notably lower than that in patients without plaque (*P* < 0.01), which is consistent with the findings of Timms et al. A previous study found that supplementation with vitamin D3 could ease the formation and fracture of plaque in patients with vitamin D deficiency [25]. Moreover, the serum 25(OH)D level in the blood glucose fluctuation group was considerably lower than that in the glucostasis group (*P* < 0.05). The severity of lower extremity angiostenosis was positively correlated with age, smoking, and HbA1c, CRP, and LDL-C levels (*r* = 0.42, 0.48, 0.65, 0.68, and 0.55, respectively, all *P* < 0.05), whereas it was negatively correlated with 25(OH)D concentration (*r* = −0.269, *P* < 0.05).

Endothelial cell dysfunction is intimately associated with the incidence and progression of AS. Vitamin D is capable of protecting endothelial function via multiple pathways. The VDR signaling system participates in regulating the incidence and development of AS through various mechanisms, such as improving endothelial function, inhibiting the formation of foam cells, suppressing vascular smooth muscle cell proliferation, and protecting blood vessels [26, 27].

This study had limitations of a single center and a small sample size. These limitations require the cooperation of the current research center with other centers to enlarge the sample size in the future.

Taken together, these results indicate that serum 25(OH)D acts as a protective factor against lower extremity vascular complications in T2DM patients. Moreover, we preliminarily discuss the possibility of using 25(OH)D levels as a predictor of preclinical lower extremity AS in T2DM patients. The findings of this study deepen our understanding of the nonclassic effect of vitamin D and provide novel clues for the prevention and treatment of lower extremity vascular complications in T2DM patients.

## Figures and Tables

**(a):** 

Group	*n* (male/female)	Age (years)	BMI (kg/m^2^)	HbA1C (%)	Fasting plasma glucose (mmol/l)
NGR	12/18	52 ± 10	23.6 ± 5.8	5.6 ± 0.5	5.1 ± 0.9
T2DM	293/297	53 ± 17	25.2 ± 8.6^∗^	8.6 ± 1.4^∗^	9.2 ± 2.8^∗^

**(b):** 

Group	2 h postprandial plasma glucose (mmol/l)	SBP (mmHg)	DBP (mmHg)	Urea (mmol/l)	Cr (*μ*mol/l)	UA (*μ*mol/l)
NGR	7.2 ± 1.6	132 ± 18	76 ± 8	4.6 ± 0.8	60 ± 13	352 ± 70
T2DM	13.5 ± 4.0^∗^	135 ± 16	80 ± 10	5.2 ± 0.9	63 ± 15	360 ± 76

**(c):** 

Group	Fasting insulin (mIU/l)	TGs (mmol/l)	TC (mmol/l)	LDL-c (mmol/l)	HDL-c (mmol/l)
NGR	12.0 ± 3.8	2.1 ± 0.5	4.2 ± 0.8	2.7 ± 0.8	1.4 ± 0.3
T2DM	14.1 ± 3.6^∗^	3.9 ± 0.8^∗^	5.6 ± 1.1^∗^	2.6 ± 0.5	1.3 ± 0.4

**(d):** 

Group	ALT (U/l)	AST (U/l)	*γ*-GGT (U/l)
NGR	41 ± 10	35 ± 12	55 ± 14
T2DM	43 ± 8	38 ± 8	80 ± 15^∗^

^∗^ *P* < 0.05 vs. NGR; values are shown as the mean ± standard deviation. NGR: normal glucose regulation; T2DM: type 2 diabetes mellitus alone.

**(a):** 

Group	*n* (male/female)	Age (years)	BMI (kg/m^2^)	HbA1C (%)	Fasting plasma glucose (mmol/l)
NGR	12/18	52 ± 10	23.6 ± 5.8	5.6 ± 0.5	5.1 ± 0.9
T2DM	227/233	54 ± 18	24.2 ± 3.9^a^	6.6 ± 0.8^a^	5.8 ± 0.4
T2DM-V(Mi)	27/26	53 ± 18	24.3 ± 6.2^b^	7.2 ± 1.9^ab^	8.2 ± 3.7^ab^
T2DM-V(Mo)	22/18	50 ± 17	25.6 ± 8.4^abc^	7.9 ± 2.8^ab^	7.8 ± 2.8^abc^
T2DM-V(Se)	17/20	52 ± 16	25.6 ± 8.4^abc^	8.6 ± 3.0^abcd^	10.6 ± 4.2^abcd^

**(b):** 

Group	2 h postprandial plasma glucose (mmol/l)	SBP (mmHg)	DBP (mmHg)	TG (mmol/l)	TC (mmol/l)	LDL-c (mmol/l)	HDL-c (mmol/l)
NGR	7.2 ± 1.6	132 ± 18	76 ± 8	2.1 ± 0.5	4.2 ± 0.8	2.7 ± 0.8	1.4 ± 0.3
T2DM	10.2 ± 5.5^a^	136 ± 18	74 ± 9	3.2 ± 0.6^a^	4.8 ± 0.9^a^	2.6 ± 0.7	1.3 ± 0.6
T2DM-V(Mi)	12.8 ± 4.6^a^	136 ± 16	82 ± 7	2.2 ± 1.2^ab^	4.6 ± 1.2^ab^	2.5 ± 0.6	1.3 ± 0.3
T2DM-V(Mo)	16.5 ± 6.2^abc^	130 ± 15	80 ± 12	3.1 ± 1.3^abc^	4.8 ± 0.8^ac^	2.7 ± 0.8	1.3 ± 0.4
T2DM-V(Se)	18.9 ± 8.4^abcd^	130 ± 18	80 ± 9	4.9 ± 2.1^abcd^	5.9 ± 1.5^abcd^	2.6 ± 0.3	1.4 ± 0.5

^a^ *P* < 0.05 vs. NGR, ^b^*P* < 0.05 vs. T2DM, ^c^*P* < 0.05 vs. T2DM-V(Mi), ^d^*P* < 0.05 vs. T2DM-V(Mo).

**Table 3:** Multivariable logistic regression analysis of the integration of lower extremity macroangiopathy.

Variable	*β*	SE (*β*)	Wald	*t*	*P*	OR	95% CI
Sex	1.039	0.767	3.46	0.87	0.733	0.415	2.71-3.10
Age	0.054	0.134	4.60	1.89	0.276	1.086	0.83-1.25
Disease course	0.043	0.306	3.80	3.42	0.485	1.040	1.03-1.42
BMI	0.043	0.895	11.75	4.29	0.383	2.702	1.68-3.37
Medication	0.123	0.089	12.2	5.02	0.278	1.718	2.75-6.05
HbA1c (%)	0.685	0.715	6.25	2.68	0.019	1.021	1.28-2.06
25(OH)D (ng/ml)	-2.49	0.617	26.81	2.68	0.020	0.772	0.69-0.86

## Data Availability

All data used for analysis in this study are available from the corresponding author on request.
